# Supplementary material for: A strategy to incorporate prior knowledge into correlation network cutoff selection
Source: Nat Commun. 2020 Oct 14;11:5153. doi: 10.1038/s41467-020-18675-3 (PMC7560866; doi:10.1038/s41467-020-18675-3)
Supplement: Supplementary file 3 — Reporting Summary [file 41467_2020_18675_MOESM3_ESM.pdf]

nature research

Corresponding author(s): Jan Krumsiek

Last updated by author(s): Aug 3, 2020

Reporting Summary

Nature Research wishes to improve the reproducibility of the work that we publish. This form provides structure for consistency and transparency in reporting. For further information on Nature Research policies, see our Editorial Policies and the Editorial Policy Checklist.

Statistics

For all statistical analyses, confirm that the following items are present in the figure legend, table legend, main text, or Methods section.

n/a	Confirmed	
☐	☒	The exact sample size (n) for each experimental group/condition, given as a discrete number and unit of measurement
☐	☒	A statement on whether measurements were taken from distinct samples or whether the same sample was measured repeatedly
☐	☒	The statistical test(s) used AND whether they are one- or two-sided<br>*Only common tests should be described solely by name; describe more complex techniques in the Methods section.*
☐	☒	A description of all covariates tested
☐	☒	A description of any assumptions or corrections, such as tests of normality and adjustment for multiple comparisons
☐	☒	A full description of the statistical parameters including central tendency (e.g. means) or other basic estimates (e.g. regression coefficient) AND variation (e.g. standard deviation) or associated estimates of uncertainty (e.g. confidence intervals)
☐	☒	For null hypothesis testing, the test statistic (e.g. F, t, r) with confidence intervals, effect sizes, degrees of freedom and P value noted<br>*Give P values as exact values whenever suitable.*
☒	☐	For Bayesian analysis, information on the choice of priors and Markov chain Monte Carlo settings
☒	☐	For hierarchical and complex designs, identification of the appropriate level for tests and full reporting of outcomes
☐	☒	Estimates of effect sizes (e.g. Cohen's d, Pearson's r), indicating how they were calculated

Our web collection on statistics for biologists contains articles on many of the points above.

Software and code

Policy information about availability of computer code

Data collection	No software used for data collection.
Data analysis	All statistical analysis has been performed with R version 4.0.1 and Rstudio version 1.3.959. The GeneNet algorithm implementation was taken from the GeneNet R package (version 1.2.15). R code to reproduce the main results of the manuscript is available at https://github.com/krumsieklab/CutoffOpt.

For manuscripts utilizing custom algorithms or software that are central to the research but not yet described in published literature, software must be made available to editors and reviewers. We strongly encourage code deposition in a community repository (e.g. GitHub). See the Nature Research guidelines for submitting code & software for further information.

Data

Policy information about availability of data

All manuscripts must include a data availability statement. This statement should provide the following information, where applicable:

- Accession codes, unique identifiers, or web links for publicly available datasets
- A list of figures that have associated raw data
- A description of any restrictions on data availability

Preprocessed glycan concentrations, as well as values corrected for age and gender, are available from the figshare database with the DOI: 10.6084/m9.figshare.5335861. The metabolomics data cohort are available upon request and after compliance with the policies and procedures of Weill Cornell Medicine-Qatar and Qatar National Research Fund for data sharing. Requests can be submitted to Hassen Al-Amin at haa2019@qatar-med.cornell.edu.
The transcriptomics data are available online through the TCGA Research Network portal at https://xenabrowser.net/datapages/?dataset=TCGA.PANCAN12.sampleMap%2FPanCan12.3602-corrected-v3_syn1715755&host=https%3A%2F%2Flegacy.xenahubs.net&addHub=https%3A%2F%2Flegacy.xenahubs.net&removeHub=https%3A%2F%2Fxena.treehouse.gi.ucsc.edu%3A443. STRING PPI network (version 10.5) was downloaded from https://

version-10-5.string-db.org/cgi/download.pl. CORUM protein complexes were taken from the ganet R package (version 2.4.2). Reactome pathways were obtained through the graphite Rpackage (version 1.26.0).

Field-specific reporting

Please select the one below that is the best fit for your research. If you are not sure, read the appropriate sections before making your selection.

[x] Life sciences   [ ] Behavioural & social sciences   [ ] Ecological, evolutionary & environmental sciences

For a reference copy of the document with all sections, see nature.com/documents/nr-reporting-summary-flat.pdf

Life sciences study design

All studies must disclose on these points even when the disclosure is negative.

Sample size	No new data were generate for this study.
Data exclusions	No data were excluded in this study.
Replication	Glycomics statistical findings have been replicated across four independent cohorts. All the analyses yielded comparable results.
Randomization	All molecular data were corrected for age and gender of participants. Metabolomics data were additionally corrected for the body mass index of the individuals, while transcriptomics data were corrected for cancer type.
Blinding	No new data were generated for this study.

Reporting for specific materials, systems and methods

We require information from authors about some types of materials, experimental systems and methods used in many studies. Here, indicate whether each material, system or method listed is relevant to your study. If you are not sure if a list item applies to your research, read the appropriate section before selecting a response.

Materials & experimental systems

n/a	Involved in the study	
[x]	[ ]	Antibodies
[x]	[ ]	Eukaryotic cell lines
[x]	[ ]	Palaeontology and archaeology
[x]	[ ]	Animals and other organisms
[x]	[ ]	Human research participants
[x]	[ ]	Clinical data
[x]	[ ]	Dual use research of concern

Methods

n/a	Involved in the study	
[x]	[ ]	ChIP-seq
[x]	[ ]	Flow cytometry
[x]	[ ]	MRI-based neuroimaging
